# Feasibility of Laparoscopic Radical Colpectomy in Locally Advanced Vaginal Cancer: A Case Report and Literature Review

**DOI:** 10.3390/jcm15010385

**Published:** 2026-01-05

**Authors:** Davut Dayan, Hannes Endres, Stefan Lukac, Wolfgang Janni, Florian Ebner, Mandana Shirin Khodawandi, Jasmina Veta Darkovski

**Affiliations:** 1 Department of Obstetrics and Gynecology, Ulm University Hospital, 89075 Ulm, Germany; stefan.lukac@uniklinik-ulm.de (S.L.); wolfgang.janni@uniklinik-ulm.de (W.J.); f.ebner@adk-gmbh.de (F.E.); mandana.khodawandiyazdi@uniklinik-ulm.de (M.S.K.); jasmina.vetadarkovski@uniklinik-ulm.de (J.V.D.); 2 Department of Obstetrics and Gynecology, University Medical Center Freiburg, 79106 Freiburg, Germany; hannes.endres@uniklinik-freiburg.de; 3 Department of Obstetrics and Gynecology, Alb-Donau Klinikum Ehingen, 89584 Ehingen (Donau), Germany

**Keywords:** vaginal carcinoma, vulvar carcinoma, minimally invasive surgery, laparoscopic colpectomy, laparoscopic radical hysterectomy

## Abstract

**Objectives**: Due to the rarity of primary vaginal carcinoma, standardized treatment approaches are limited. Radical surgery is rare, especially in advanced stages. This report evaluates the feasibility, technical aspects and outcomes of laparoscopic en bloc resection in advanced vaginal carcinoma. **Case presentation**: A 67-year-old woman presented with pain and vaginal bleeding. Clinical examination revealed a stenosing vaginal tumour up to 2 cm above the introitus, extending to the urethra and right vulva. Biopsies confirmed invasive squamous cell carcinoma with VAIN/VIN III. Imaging revealed enlarged pelvic lymph nodes, but no distant metastases. **Methods**: The surgical procedure comprised laparoscopic en bloc resection, including bilateral pelvic lymphadenectomy, radical hysterectomy with bilateral salpingo-oophorectomy, and total vaginal excision down to the pelvic floor. Additionally, inguinal bilateral ICG-guided sentinel lymph node dissection, vulvectomy with clitoral preservation, and partial urethral resection were performed, followed by transvaginal specimen removal. Vaginal closure was achieved via combined transvaginal and laparoscopic pelvic floor reconstruction. The postoperative course was uneventful, with early recovery of urinary and bowel function. Final histology confirmed complete tumor resection with clear margins (pT3, pN0, L0, V0, Pn0, R0). Functional outcomes remained excellent, with no recurrence or functional impairment at one-year follow-up. **Conclusions**: Laparoscopic en bloc resection appears to be a feasible option for selected patients with locally advanced vaginal carcinoma, enabling complete tumour removal with preservation of pelvic floor function and resulting in favourable postoperative and oncological outcomes.

## 1. Introduction

Primary vaginal carcinoma is one of the rarest malignant neoplasms of the female genital tract, accounting for only 1–2% of all gynecologic cancers [1,2]. Only about 10% of vaginal malignancies are of primary vaginal origin, whereas the majority represent metastases from adjacent organs such as the cervix, endometrium, vulva, or rectum. The diagnosis of primary vaginal carcinoma necessitates the exclusion of any current malignancy—or one that has occurred within the preceding five years—in these regions, unless a distinctly different histologic subtype is evident [3].

The disease predominantly affects women over 60 years of age, with a mean age of approximately 68 years, though it can also occur in younger patients [2,4,5,6]. In nearly two-thirds of cases, the upper third of the vagina is involved [1,4,7]. Major risk factors include persistent infection with high-risk human papillomavirus (HPV), repeated mechanical trauma, and chronic inflammation [1,6,7]. Histopathologically, squamous cell carcinoma predominates, accounting for approximately 85% of cases, followed by adenocarcinoma [2,6]. Other histologic variants such as malignant melanoma, neuroendocrine tumors, and papillary squamous cell carcinoma are far less common [6,8].

Owing to the rarity of this disease and limited data availability, no standardized therapeutic protocol has been established. Treatment must therefore be individualized according to tumor stage, location, comorbidities, and the patient’s general condition. Radical surgical procedures are rarely performed, as they are associated with considerable physical and psychological burden, higher complication rates, and technically demanding R0 resections [9,10,11,12]. Consequently, radiation therapy is considered the standard of care in most centers, particularly for advanced stages [13,14].

Nevertheless, several studies suggest a survival advantage with primary surgical treatment compared with radiotherapy alone, especially in early-stage disease [2,4,5,15,16,17,18]. Surgery offers improved local tumor control and the possibility of curative salvage options in the event of recurrence—an approach rarely feasible after definitive radiotherapy. In younger patients, partial radical vaginectomy with reconstruction can further contribute to sexual function preservation [4]. In addition, recent reports have highlighted the potential role of neoadjuvant chemotherapy followed by radical surgery in locally advanced tumors to reduce tumor volume, improve resectability, and optimize long-term oncologic outcomes [13,19].

Over the past decade, minimally invasive surgery has emerged as a promising alternative to open approaches in the management of vaginal carcinoma. Several case reports and small series have described laparoscopic procedures as oncologically safe and technically feasible options in carefully selected early-stage patients [2,20,21].

This report presents the methodology, feasibility, and oncologic outcomes of laparoscopically assisted radical colpectomy combined with radical hysterectomy, vulvectomy, and inguinal and pelvic lymphadenectomy. To our knowledge, this is the first documented case of advanced pT3 vaginal carcinoma successfully treated with a multimodal minimally invasive approach. The case particularly emphasizes the technical feasibility, oncologic safety, perioperative outcomes, and functional preservation of the pelvic floor.

## 2. Presentation of the Case

A 67-year-old Caucasian woman presented with gradually worsening sitting pain and vaginal bleeding. She was in good general condition (ECOG “Eastern Cooperative Oncology Group” performance status 0) and had a body mass index (BMI) of 21.7 kg/m^2^. The ECOG performance status is a widely used measure of functional capacity and ranges from 0 (fully active) to 5 (death), as follows:

- ECOG 0: Fully active; able to carry on all pre-disease activities without restriction.
- ECOG 1: Restricted in physically strenuous activity but ambulatory; able to carry out light or sedentary work.
- ECOG 2: Ambulatory and capable of all self-care but unable to carry out any work activities; up and about >50% of waking hours.
- ECOG 3: Capable of only limited self-care; confined to bed or chair > 50% of waking hours.
- ECOG 4: Completely disabled; cannot carry on any self-care; totally confined to bed or chair.
- ECOG 5: Dead.

Due to menopausal symptoms, she had received combined hormone replacement therapy until the age of 63 years. Her medical history was notable for Raynaud’s syndrome, with no other significant comorbidities or regular medications. There was no relevant family history of malignancy, except for one relative with breast cancer.

A long-standing lichen planus had been diagnosed several years earlier and was successfully managed with low-dose topical glucocorticoid therapy (clobetasol). One year prior to presentation, a biopsy was performed due to progressive vaginal stenosis and condylomatous changes, revealing lower genital tract intraepithelial neoplasia (LGTIN), classified as vaginal intraepithelial neoplasia grade II (VAIN II) and vulvar intraepithelial neoplasia grade II (VIN II). At that time, no HPV infection was detected.

**Figure 1:**
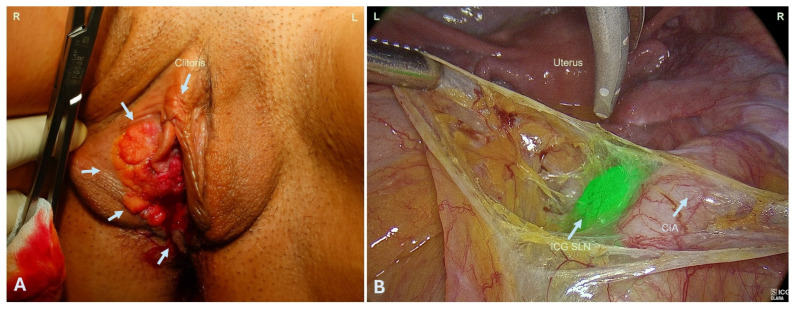
(**A**) Intraoperative presentation of vaginal carcinoma infiltrating into the right vulva. (**B**) Laparoscopic imaging of the ICG-labeled sentinel lymph nodes iliaca communis on the right. CIA: common iliac artery, ICG SLN: Indocyanine green sentinel lymph node.

Clinical examination revealed almost complete obliteration of the vagina, with only a small opening remaining due to a stenosing, partially necrotic, and bleeding tumor. The lesion infiltrated the distal urethra and extended to both labia and the right vulva (Figure 1A). Speculum examination was not feasible due to severe pain. Palpation demonstrated vaginal stenosis approximately two centimeters proximal to the introitus with thickened mucosa, preventing complete assessment. Transvaginal sonography was therefore omitted.

During an examination under anesthesia with mapping biopsies, histopathological analysis confirmed an HPV-associated squamous cell carcinoma (SCC) involving both the vagina and vulva, with an invasion depth of approximately 0.2 cm in each site. Immunohistochemistry demonstrated p16 positivity, p53 wild-type expression, and a Ki-67 proliferation index of approximately 50%.

At diagnosis, the primary tumor could not be definitively assigned to either the vagina or vulva. Preoperative staging with computed tomography (CT) of the thorax and abdomen and magnetic resonance imaging (MRI) of the pelvis revealed several slightly enlarged inguinal and pelvic lymph nodes, without definitive evidence of metastatic disease. No distant metastases were identified.

The case was subsequently discussed in an interdisciplinary tumor board. Given the pronounced tumor-associated vaginal stenosis and histologically confirmed malignancy involving both the vagina and vulva, both curative surgical therapy and primary radiotherapy were considered as potential treatment options.

After comprehensive counseling on the diagnosis and therapeutic alternatives, including open and minimally invasive surgery as well as radiotherapy, the patient opted for minimally invasive surgery with curative intent. She explicitly requested preservation of the clitoris and declined vaginal reconstruction. The treatment plan was developed in accordance with her preferences, and written informed consent was obtained prior to surgery.

## 3. Surgical Technique

Preoperatively, radioactive labeling of the inguinal sentinel lymph nodes was performed. Laparoscopic colpectomy was carried out under general anesthesia in the lithotomy position. Perioperative antibiotic prophylaxis consisted of cefuroxime 2.5 g intravenously every 4 h and metronidazole 500 mg intravenously every 8 h.

Intraoperatively, an exophytically growing tumor was identified. Tumor infiltration had almost completely obliterated the vaginal canal, allowing digital palpation only to a depth of approximately 2 cm. The lesion extended from the vagina across the vulva, involving the entire right labium minus and the adjacent right labium majus, and ran ventrally to terminate just below the clitoris. On the left side, it infiltrated approximately half of the labium minus.

Under sterile conditions, a 14 Ch transurethral Foley catheter was inserted. Subsequently, 4 mL of indocyanine green (ICG; Verdye^®^, 25 mg, Diagnostic Green GmbH, Aschheim-Dornach, Germany) at a concentration of 2.5 mg/mL (prepared by dissolving 25 mg ICG in 20 mL sterile water) was injected into the vulvar and vaginal tissues surrounding the tumor to facilitate sentinel lymph node mapping.

The laparoscopic approach was initiated using a closed technique with Veress needle insertion at the umbilicus, followed by CO_2_ insufflation to an intra-abdominal pressure of 15 mmHg. A 10 mm, 30° laparoscope was utilized, and three additional trocars were placed under direct vision (5 mm each in the right and left lower quadrants and a 12 mm suprapubic trocar).

Exploratory laparoscopy revealed unremarkable findings of the upper abdominal organs and peritoneal surfaces. The gallbladder appeared distended with a thickened wall, consistent with preoperative radiologic findings of adenomyotic hypertrophy.

Using ICG fluorescence imaging, sentinel lymph nodes were identified along the right external and common iliac arteries and excised for intraoperative frozen section analysis (Figure 1B). Because suspicious pelvic lymph nodes were observed radiologically and intraoperatively, a systematic radical pelvic lymphadenectomy was performed as previously discussed and consented to by the patient.

After bipolar coagulation, the round ligaments and infundibulopelvic ligaments were transected bilaterally. Both ureters were dissected retroperitoneally and exposed down to their junction with the uterine arteries. The uterine arteries were coagulated and divided at their origin from the internal iliac artery. The urinary bladder was mobilized bluntly and sharply to the level of the anterior vaginal wall, and the ureters were further dissected to their intramural course within the bladder wall.

**Figure 2:**
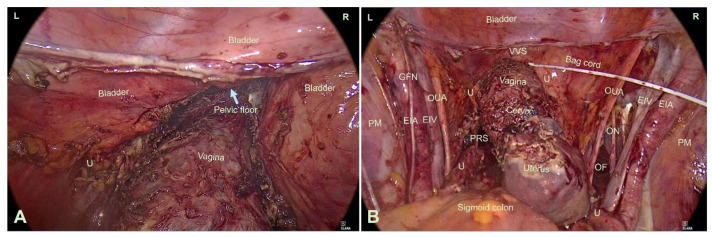
(**A**) Intraoperative imaging of the pelvic floor from the front after dissection and distancing of the bladder. (**B**) Intraoperative presentation of the specimen (uterus with vagina) deposited to the pelvic floor after dissection and distancing of the urinary bladder and rectum. EIA: ex-ternal iliac artery, EIV: External iliac vein, GFN: genitofemoral nerve, L: Left, PM: psoas major muscle, PRS: pararectal space, U: Ureter, OF: obturator fossa, ON: obturator nerve, OUA: obliteral umbilical artery, R: right, VVS: Vesico Vaginal Space.

For improved visualization, both adnexa were detached from the uterus, placed in an endoscopic retrieval bag, and temporarily stored in the right upper abdomen. The rectum was then separated from the posterior vaginal wall, opening the bilateral Latzko spaces. The uterosacral and parametrial ligaments, including the cardinal structures, were coagulated and divided. Under transvaginal digital guidance, both the bladder and rectum were mobilized entirely and separated from the vagina down to the level of the pelvic floor and introitus. The vesical branches of the inferior hypogastric plexus and the lateral portions of the vesicovaginal ligaments were preserved in a nerve-sparing manner (Figure 2A,B).

Because of intraoperative suspicion of adenomyotic hypertrophy of the gallbladder and macroscopic abnormalities consistent with preoperative imaging, a laparoscopic cholecystectomy was performed by the visceral surgeon without complications. The gallbladder was placed in a retrieval bag and temporarily left in the right upper abdomen.

**Figure 3:**
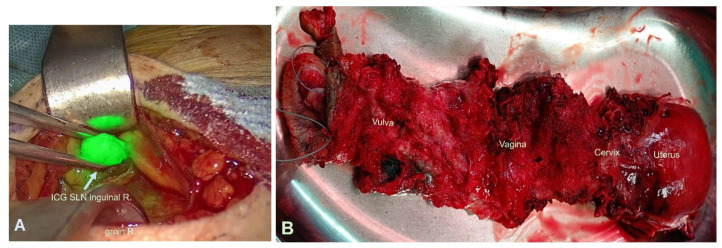
(**A**) Intraoperative visualisation of the ICG-SLN in the right groin. (**B**) End-block specimen of the vulva, distal urethra, vagina and uterus.

Following completion of the laparoscopic phase, bilateral inguinal sentinel lymph node dissection was performed using ICG guidance (Figure 3A). Frozen section analysis revealed three right-sided and two left-sided sentinel lymph nodes to be free of tumor, so radical inguinal lymphadenectomy was not undertaken. The procedure then proceeded transvaginally. At the patient’s explicit request, the clitoris was preserved, whereas the tumor-infiltrated vulva was excised with a safety margin of at least one centimeter. The resection included the entire affected vulvar region with subcutaneous fat tissue and approximately one centimeter of the distal urethra. Vaginal dissection continued up to the level previously mobilized laparoscopically.

The specimen—including the vulva, distal urethra, vagina, and uterus—was removed en bloc (Figure 3B). The adnexa, previously detached and stored in the retrieval bag, as well as the gallbladder, were also removed transvaginally. The integrity of the rectum and urinary bladder was confirmed intraoperatively using an indigo carmine dye test.

### 3.1. Pelvic Floor Closure

Closure of the pelvic floor was performed using a combined vaginal and laparoscopic approach. Vaginally, a multilayer closure was achieved by mobilizing the remaining vulvar tissue and approximating it to the levator muscles with interrupted Vicryl sutures (0 or 2-0). Particular care was taken to ensure correct, tension-free reconstruction of the urethra. A vaginal drain was placed for postoperative drainage.

**Figure 4:**
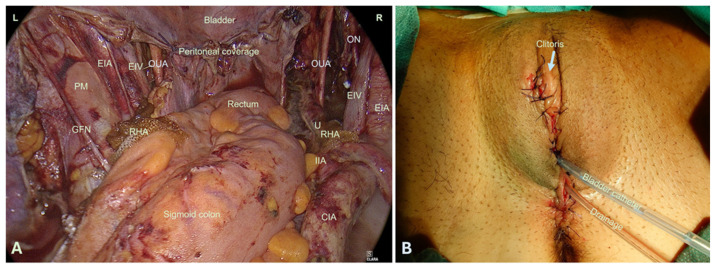
(**A**) Laparoscopic imaging of the surgical site at the end of the operation, after the vagina and peritoneum have been closed. For adhesion prophylaxis, the ureters were covered with an absorbable hemostatics. (**B**) Imaging of the vulva at the end of the operation with the urinary catheter lying down and drainage. CIA: common iliac artery, EIA: external iliac artery, EIV: External iliac vein, GFN: genitofemoral nerve, IIA: Internal iliac artery, L: Left, PM: psoas major muscle, U: Ureter, ON: obturator nerve, OUA: Oliteral umbilical artery, R: right, RHA: resorbable hemostatic agent.

Laparoscopically, the stumps of the uterosacral and round ligaments were approximated and sutured where feasible using interrupted 0-Vicryl sutures. The peritoneum was then closed over the defect with a continuous 2-0 Vicryl suture (Figure 4A). All wound closures were performed with meticulous attention to tension-free adaptation of each tissue layer. Finally, a transurethral silicone Foley catheter was inserted for urinary diversion (Figure 4B).

### 3.2. Postoperative Findings, Surgical Outcome and Recovery

The intraoperative course was uneventful. The total operative time was 600 min, with an estimated blood loss of 300 mL. No intraoperative or postoperative transfusion was required. The patient resumed oral intake immediately, and no further antibiotics were administered. The transurethral catheter was removed on postoperative day 2, after which spontaneous voiding occurred without residual urine. The first bowel movement occurred on postoperative day 2 without discomfort.

Recovery was clinically uneventful. Because of increased psychosomatic support needs, hospital discharge was delayed until postoperative day 11. At discharge, the patient was in good general condition and reported subjective well-being.

Histopathologic examination of the completely resected specimen (vulva, vagina, and uterus; measuring 14.8 × 5.3 × 3.0 cm) revealed vaginal carcinoma that had infiltrated the entire length of the vagina, extending to the vulva and cervical surface. The pathological staging was pT3, pN0 (right: 13 lymph nodes, including three inguinal sentinel nodes; left: 27 lymph nodes, including two inguinal sentinel nodes), L0, Pn0, V0, R0 and G2. All resection margins were histologically tumor-free.

An uncomplicated urinary tract infection diagnosed on the day of discharge was successfully treated with a three-day course of oral antibiotics.

During the four-week follow-up, the patient was asymptomatic, with no urinary or bowel dysfunction. Vaginal examination showed a non-irritated, well-healed surgical field without infection, wound dehiscence, or recurrence. At 12 months, she remained symptom-free with no clinical or radiologic evidence of local or systemic recurrence.

## 4. Materials and Methods

This review was conducted as a focused review involving a structured literature search. Relevant publications were identified by searching PubMed/MEDLINE, Embase and the Cochrane Library using predefined keywords and phrases related to minimally invasive radical surgery for primary vaginal cancer. These included: ‘laparoscopic surgery for primary vaginal cancer’ (n = 72), ‘colpectomy for vaginal carcinoma’ (n = 25), ‘laparoscopic radical hysterectomy and vaginectomy’ (n = 16), and ‘laparoscopic radical vaginectomy for vaginal carcinoma’ (n = 11).

Eligible publications included case reports, case series, and observational studies that reported on laparoscopic or robot-assisted radical colpectomy or vaginectomy for malignant primary vaginal diseases. Studies were excluded if they dealt with benign indications, malignancies other than primary vaginal cancer, non-endoscopic procedures or tumours that did not originate in the vagina. Articles not published in English were also excluded.

After removing 12 duplicates, nine non-English records were excluded. The full texts of the remaining 14 articles were reviewed for eligibility. Nine additional studies were excluded based on the predefined criteria after full-text review. Ultimately, five studies were included in the final analysis (see Table 1).

## 5. Discussion

Primary vaginal carcinoma is rare, and there is limited evidence, so no standardized treatment algorithms exist, and management must be highly individualized. The variability in tumor location, the complex pelvic anatomy, and the involvement of adjacent organs make it challenging to define a uniform treatment strategy. Where feasible, primary surgery with curative intent is generally considered the preferred treatment; in this context, our case demonstrates the potential of laparoscopically assisted radical surgery as a curative option, even for advanced vaginal carcinoma.

The incidence of primary vaginal carcinoma peaks in the sixth and seventh decades of life. The most common clinical manifestations are postcoital and/or postmenopausal vaginal bleeding, while palpable vaginal masses may indicate local extension and result in urinary or gastrointestinal symptoms. Pelvic pain is reported by only a minority of patients, and up to 20% remain asymptomatic. Vaginal cancer is frequently detected in the context of cervical cancer screening or during routine pelvic examination [2,3,6,7,25,26]. Most cases are diagnosed at FIGO stage I [6]. In our patient, the leading symptoms were severe pain and vaginal bleeding. Nearly complete vaginal stenosis, which prevented sexual intercourse, is an uncommon presentation that has rarely been reported.

Due to the rarity of this malignancy, standardized therapeutic protocols are lacking. Current evidence is based almost exclusively on retrospective studies and small case series, as prospective randomized trials are absent. Consequently, treatment recommendations primarily derive from established guidelines for cervical and anal carcinomas [2,3,4,6]. Management must therefore be individualized, considering tumor location, size, stage, and patient preference [2,4,7].

Prognosis deteriorates significantly with advanced FIGO stage, primarily due to comorbidities, lower resection rates, and limitations in radiation dose [6]. Furthermore, tumor size correlates negatively with oncological outcome: in a multivariate analysis, Hellman et al. identified lesions > 4 cm as an independent risk factor for reduced survival (HR 2.1) [27], and Wolfson et al. showed that a size cut-off of 2 cm was already associated with a decline in 5-year overall survival, from 79.2% (≤2 cm) to 66.1% (>2 cm) in stage I, and from 80.9% to 51.2% in stage II [28]. Similarly, Huang et al. reported a 1.62-fold increase in mortality risk for tumors > 4 cm [6].

The complex pelvic anatomy, with the vagina in close proximity to the bladder, rectum, major vessels and autonomic nerves, makes radical resection technically challenging, especially for tumors measuring more than 2 cm or located in the middle of the vagina [2,3,4,7,29]. Nevertheless, primary surgery can achieve excellent outcomes for lesions ≤ 2 cm in the upper third of the vagina (FIGO I–II), with reported 5-year survival rates of up to 100% [2,4,7,29]. In advanced stages, however, surgical resection—especially via open approaches—is frequently limited by restricted exposure and organ infiltration and is associated with increased morbidity; accordingly, primary radio(chemo)therapy remains the standard of care [3,7,8].

However, advances in conventional laparoscopic and robot-assisted surgery, as well as experience from radical interventions in cervix/endometrial cancer and deeply infiltrating endometriosis, have enabled new minimally invasive resection strategies. In early-stage disease, case series report lower complication rates, faster convalescence, and oncologic outcomes comparable to open surgery [2,20,22,23,30]. Laparoscopic techniques, including laparoscopic closure of the vaginal cuff, are associated with reduced postoperative morbidity and lower rates of cuff dehiscence and infection, which is particularly relevant in oncologic surgery [31].

Magrina et al. first demonstrated the technical feasibility of a minimally invasive radical approach to treating vaginal carcinoma. They performed laparoscopic radical parametrectomy with partial vaginectomy and pelvic and para-aortic lymphadenectomy on a patient who had previously undergone radiotherapy for FIGO IIB adenocarcinoma of the upper vaginal stump. There was no significant perioperative morbidity, and the patient experienced only transient urinary retention [22]. Subsequent studies have confirmed mainly that laparoscopic radical procedures can achieve complete tumor resection with an acceptable perioperative risk profile in selected early-stage cases. Ling et al. reported on four sexually active patients with FIGO stage I vaginal carcinoma in the upper or upper two-thirds of the vagina who underwent laparoscopic radical hysterectomy with vaginectomy and reconstruction. R0 resection was achieved in all cases, with moderate operative times and blood loss, a short recovery period, and recurrence-free survival at a median of 46 months. Sexual function was also preserved [2]. Li et al. extended these findings to 12 patients (predominantly FIGO I and one FIGO II), including primary and recurrent tumors. They were treated with laparoscopic nerve-sparing radical vaginectomy (with pelvic/paraaortic lymphadenectomy in selected cases). The researchers reported minimal blood loss and no intraoperative complications or transfusions. All patients achieved R0 resection and experienced a rapid recovery of bladder function. There were no recurrences during a median follow-up period of 28 months [20]. Yao et al. further demonstrated that minimally invasive radical surgery can be combined with immediate peritoneal or sigmoid vaginoplasty for FIGO I tumors of the upper third of the vagina. This approach achieved R0 resection in all 12 patients and was associated with low complication rates and no recurrences at 36 months [23]. The case reported by Van Trappen et al. suggests that robotic assistance may offer technical advantages such as enhanced visualization and dexterity in the deep pelvis. This facilitates the precise resection of lower-third vaginal malignancies with acceptable blood loss and an uneventful postoperative recovery [24].

In contrast to previously published series, which predominantly include small FIGO I–II tumors of the upper vagina, our patient presented with an extensive vaginal carcinoma extending from the cervix to the vulva and causing near-complete vaginal stenosis. This precluded the use of a uterine or vaginal manipulator and substantially increased technical complexity. Yet, laparoscopically assisted radical colpectomy with radical hysterectomy, vulvectomy, and lymph node staging was completed without intra- or postoperative complications. The prolonged operating time can be attributed to the absence of a manipulator and the concomitant cholecystectomy, while the intraoperative blood loss of approximately 300 mL was within the range reported for minimally invasive series, and no transfusion was required. Vaginal reconstruction was deliberately omitted in accordance with the patient’s preference.

We conducted a literature review to assess the feasibility of minimally invasive approaches in vaginal carcinoma; the key findings are summarized in Table 1.

To our knowledge, this is the first documented case of laparoscopically assisted radical colpectomy combined with radical hysterectomy, vulvectomy, and lymphadenectomy for advanced vaginal cancer. The achievement of tumor-free resection margins, the absence of perioperative complications, and the preservation of pelvic floor function support the technical feasibility and suggest the oncological safety of a minimally invasive approach even in anatomically complex, advanced disease.

Nevertheless, despite these and other encouraging reports, substantial evidence gaps remain regarding the role of laparoscopy in advanced vaginal carcinoma. Randomized trials and prospective data on oncological outcomes are lacking, and no firm recommendations can currently be issued. In view of the generally poor prognosis, radiotherapy remains the standard of care, although individualized radical surgery, including minimally invasive techniques, may be considered in carefully selected patients. Further studies are required to define more precisely the role of minimally invasive procedures in advanced stages.

## 6. Conclusions

Based on this case, laparoscopically assisted vaginal radical hysterectomy, vaginectomy, and vulvectomy with lymphadenectomy appear to be safe and effective, although the follow-up time was short (~12 months). Accordingly, it may be a feasible alternative to chemoradiotherapy for advanced vaginal cancer patients. Nevertheless, this represents a single case; therefore, extensive clinical research is necessary to further elucidate the benefits and risks of this procedure within this patient population.

## Figures and Tables

**Table 1:** Oncological and surgical characteristics.

Study	Center	No. of Patients	Age (y)	Localisation of the Tumor	Stage of Disease (FIGO)	Type of Operation	Operative Time (m) *	Estimated Blood Loss (mL) *	Postoperative Complications	FU * (mo)
Magrina et al., 1999 [22]	United States	1	63	Upper 1/3	FIGO IIb	Preoperative RT + radical parametrectomy with vaginectomy and PALND + PLND	270	200	none	WELL
Ling et al., 2008 [2]	China	4		Upper 2/3	FIGO I	LRHV + sigmoid vaginoplasty	305 (260–350)	325 (250–400)	none	46
Li et al., 2012 [20]	China	12	52.8 * (9.7)	Upper 2/3	FIGO I (11), FIGO II (1)	LRHV or LRV + PLND/PALND	158.5 (36.7)	135.2 (62.8)	none	28
Yao et al., 2014 [23]	China	12	39–61	Upper 2/3	FIGO I	LRHV + sigmoid or peritoneal vaginoplasty	245 ± 46.4 (Peritoneal Group) 300 ± 26.5 (Sigmoid Group)	320 ± 103.7 (Peritoneal Group) 340 ± 82.7 (Sigmoid Group)	none	36
Van Trappen et al., 2023 [24]	Belgium	1	73	Lower 1/3	FIGO I	rSPLND + en bloc HV	195	300	None	4
Current study	Germany	1	67	Entire vagina with infiltration of the vulva and cervix	FIGO III	LRHV +BSO + vulvectomy + PLND + SILND	600	300	none	12

FU: follow-up time, FIGO: Federation of International Gynecology and Obstetrics, HV: hysterectomy and vaginectomy, NA: nonapplicable, m: minutes, mL: milliliter, mo: months, y: years, LRH: laparoscopic radical hysterectomy, LRHV: Laparoscopic radical hysterectomy and vaginectomy, PLND: pelvic lymph node dissection, PALND: paraaortic lymph node dissection, RT: radiotherapy, SILND: sentinel inguinal lymph node dissection rSPLND: robotic sentinel pelvic lymph node dissection. * Mean or median +/− Standard deviation.

## Data Availability

All relevant data supporting the findings of this case report are included within the article. Additional details may be made available by the corresponding author upon reasonable request. The case was presented as a poster at the ESGE 34th Annual Congress.
